# Supplementary material for: The interactome of intact mitochondria by cross-linking mass spectrometry provides evidence for coexisting respiratory supercomplexes
Source: Mol Cell Proteomics. 2017 Dec 8;17(2):216–32. doi: 10.1074/mcp.RA117.000470 (PMC5795388; doi:10.1074/mcp.RA117.000470)
Supplement: Supplemental Data [file supp_RA117.000470_133922_0_supp_23978_fzffwf.zip › spectra_annotation/mito_DR_spectra_annotation/139-2-1-1-14-1.pdf]

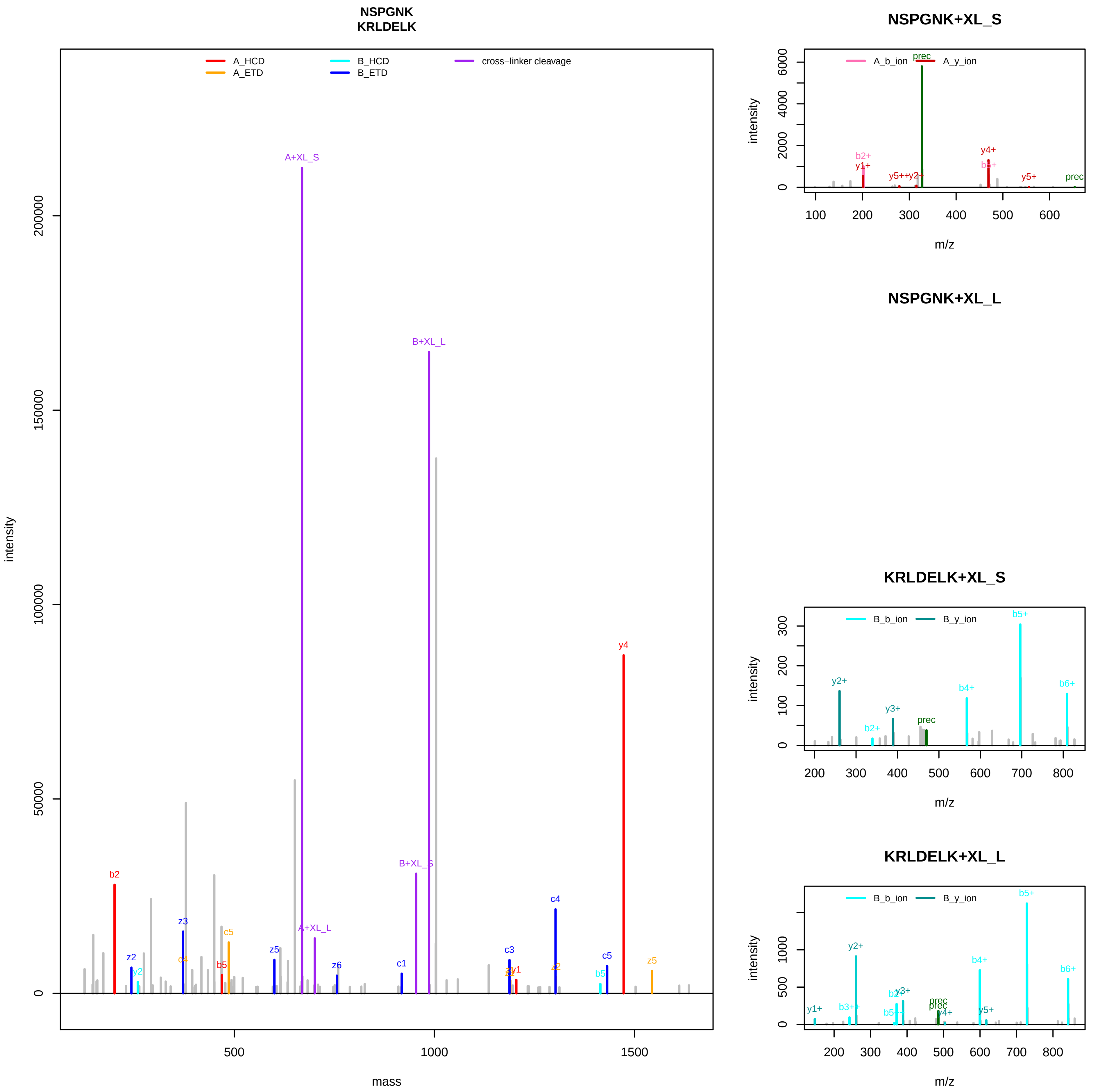
NSPGNK
KRLDELK
A_HCD
A_ETD
B_HCD
B_ETD
cross−linker cleavage
A+XL_S
B+XL_L
B+XL_S
A+XL_L
b2
z2
y2
z3
c4
b5
c5
z5
z6
c1
c3
y1
z2
c4
b5
c5
y4
z5
intensity
mass
NSPGNK+XL_S
A_b_ion
A_y_ion
prec
b2+
y1+
y5++y2+
y4+
b5+
y5+
m/z
NSPGNK+XL_L
KRLDELK+XL_S
B_b_ion
B_y_ion
b5+
y2+
b4+
b6+
y3+
b2+
KRLDELK+XL_L
y1+
b3++
b5++
y4+
y5+
